# Supplementary material for: The association of health insurance and race with treatment and survival in patients with metastatic colorectal cancer
Source: PLoS One. 2022 Feb 17;17(2):e0263818. doi: 10.1371/journal.pone.0263818 (PMC8853572; doi:10.1371/journal.pone.0263818)
Supplement: Attachment — Submitted filename: Response to Reviewers2.docx [file pone.0263818.s001.docx]

Journal Requirements

1. Please ensure that your manuscript meets PLOS ONE's style requirements, including those for file naming. The PLOS ONE style templates can be found at

<https://journals.plos.org/plosone/s/file?id=wjVg/PLOSOne_formatting_sample_main_body.pdf>

<https://journals.plos.org/plosone/s/file?id=ba62/PLOSOne_formatting_sample_title_authors_affiliations.pdf>

*Thank you for pointing out these templates for your journal formatting and styling requirements. We have revised the manuscript in order to conform to these standardized guidelines.*

2. In your Data Availability statement, you have not specified where the minimal data set underlying the results described in your manuscript can be found. PLOS defines a study's minimal data set as the underlying data used to reach the conclusions drawn in the manuscript and any additional data required to replicate the reported study findings in their entirety. All PLOS journals require that the minimal data set be made fully available. For more information about our data policy, please see <http://journals.plos.org/plosone/s/data-availability>.

"Upon re-submitting your revised manuscript, please upload your study’s minimal underlying data set as either Supporting Information files or to a stable, public repository and include the relevant URLs, DOIs, or accession numbers within your revised cover letter. For a list of acceptable repositories, please see <http://journals.plos.org/plosone/s/data-availability#loc-recommended-repositories>. Any potentially identifying patient information must be fully anonymized.

Important: If there are ethical or legal restrictions to sharing your data publicly, please explain these restrictions in detail. Please see our guidelines for more information on what we consider unacceptable restrictions to publicly sharing data: <http://journals.plos.org/plosone/s/data-availability#loc-unacceptable-data-access-restrictions>. Note that it is not acceptable for the authors to be the sole named individuals responsible for ensuring data access.

We will update your Data Availability statement to reflect the information you provide in your cover letter.

*Thank you for your comment on data availability. We apologize that we did not better explain our restrictions on that matter in our previous availability statement. The data used in our study was obtained from the National Cancer Database (NCDB), specifically the Participant User File (PUF) 2016, containing patient cases with colon and rectal cancer diagnosed in 2004-2016. The specific data available from the NCDB are used under license for the current study, and thus are not publicly available. The American College of Surgeons Participant User Data File is available at:* [*https://ncdbapp.facs.org/puf/*](https://ncdbapp.facs.org/puf/)

3. PLOS requires an ORCID iD for the corresponding author in Editorial Manager on papers submitted after December 6th, 2016. Please ensure that you have an ORCID iD and that it is validated in Editorial Manager. To do this, go to ‘Update my Information’ (in the upper left-hand corner of the main menu), and click on the Fetch/Validate link next to the ORCID field. This will take you to the ORCID site and allow you to create a new iD or authenticate a pre-existing iD in Editorial Manager. Please see the following video for instructions on linking an ORCID iD to your Editorial Manager account: <https://www.youtube.com/watch?v=_xcclfuvtxQ>

*Thank you for your comment. The corresponding author is Dr. Rebecca A. Snyder, and her updated ORCID iD number is: 0000-0002-2485-2481. Please include it in any update in the manuscript and journal as you see fit. Thank you again for your assistance in this.*

Reviewer Comments to the Author

Reviewer 1

Overall, this is a well written manuscript evaluating receipt of chemotherapy for mCRC influenced by race and insurance status using NCDB.

1. Why were other races excluded from analysis? Please provide justification for this.

*Thank you very much for your comments and for this excellent question. Initially, our intention was to compare Non-Hispanic White and Non-Hispanic Black patients only, since most previous studies have noted the most concerning disparities between these two population groups. However, per your suggestion, we have now included all races (Non-Hispanic White, Non-Hispanic Black, Hispanic, Other) in our revised analysis. In our revised manuscript, all the tables, figures, and results, have been updated accordingly with the addition of the Hispanic and Other races in the cohort.*

Reviewer 2

I appreciate the opportunity to review this paper. Mitsakos and colleagues presents a retrospective study of patients from the NCDB diagnosed with metastatic colorectal cancer from 2006-2016, and aimed to evaluate the interaction of health insurance status and race with treatment and survival. The primary outcome was receipt of chemotherapy and 3-year all-cause mortality. The found that Black patients were less likely to receive chemotherapy, had a higher 3-year mortality rate, and were less likely to receive chemotherapy even when stratified by insurance status as compared to White patients. Using their two-way interaction term analysis, they were able to show that insurance status alone did not account for the disparities in treatment and outcomes according to race. The finding that there are disparities in treatment for colorectal cancer by race is not novel, but what is novel is the finding that these disparities cannot be explained solely by insurance status or quality of insurance.
I have the following questions aimed at strengthening the manuscript.

1. Was “Receipt of chemotherapy” in the interaction analyses inclusive of palliative chemotherapy?

*Thank you for your comments and for this excellent question. According to the NCDB Participant User Data File (NCDB PUF), “Receipt of chemotherapy” includes any type of systemic chemotherapy administered to the patient at any point in time, with the exception of chemotherapy provided to control symptoms, alleviate pain, or make the patient more comfortable, which is then included in the item “Palliative Care”. Based on this definition, “Receipt of chemotherapy” in the interaction analyses is not inclusive of palliative chemotherapy.*

2. Why did the authors choose to exclude the Hispanic or Other groups of patients for this paper? It would be interesting to know if similar findings applied to these groups as well, and what the impact of insurance status on treatments and outcomes are for them.

*Thank you for this thoughtful comment. As we also responded to Reviewer 1 (above), our intention was to compare Non-Hispanic White and Non-Hispanic Black patients only, since most previous studies have noted the most concerning disparities between these two population groups. However, per your suggestion, we have now included all races (Non-Hispanic White, Non-Hispanic Black, Hispanic, Other) in our revised analysis. All the tables, figures, and results have been updated accordingly with the addition of the Hispanic and Other races in the cohort. The Hispanic subcohort appears to be numerically under-represented in this NCDB dataset in comparison to US census data, which could limit generalizability. We have discussed this potential limitation in the Discussion.*

3. What do the authors believe is the explanation for their findings that Black patients with “better” insurance (private, Medicare) are less likely to receive chemotherapy compared to White patients? The authors offer a hypothesis for their findings that under-insured groups have likely universally less access to care, thus explaining why this was not found in those insurance groups. Although, they state that the cohorts were smaller in the underinsured groups, and thus underpowered to observe a similar association with the “worse” insurance groups. What is their explanation for this finding?

*Thank you for this excellent comment which illustrates one of the major points of the study. Race, insurance status, and social determinants of health likely all play a complex role in colorectal cancer treatment delivery and mortality, and the relative contribution of each is difficult to characterize. Even when trying to isolate these factors by statistical analysis, it appears that just the improvement of health insurance status in racially disadvantaged populations cannot completely mitigate racial disparities in treatment and survival. We believe this illustrates that other underlying social determinants of health, such as general healthcare access, neighborhood segregation, or health literacy, for example, likely contribute to these disparities and warrant further exploration.*

4. Suggest adding to the discussion that although yes, the authors found that there are differences in receipt of chemotherapy based upon race, but the differences are actually small, perhaps smaller than they may have hypothesized. Because of such high numbers of patients in the NCDB, every comparison is essentially statistically significant if different by 0.1%.

*Thank you for this suggestion. It is an inherent limitation of our study, as with any retrospective study dealing with very large cohort samples, that univariate analyses can yield statistically significant differences due to a large sample size, but that these differences are not always clinically significant. For this reason, we elected to delete all p-values from Table 2 and from the univariate analysis of receipt of chemotherapy by race discussed in the Results. We hope that this allows the reader to focus on the adjusted multivariable analysis, which is more informative as it accounts for important confounding factors.*

5. Is the 3-year all cause mortality presented in Figure 2 statistically significant?

*Differences in 3-year all-cause mortality by race are statistically significant. However, as stated above, we did not report a log-rank analysis due to the large sample size and likelihood of a minor statistical significance in the absence of clinical significance. For this reason, we elected to present these findings as a Life-Table curve with the associated unadjusted all-cause mortality rates for each race.*

6. In the methods the authors state they collected data including surgery, and combination chemo+surgery, but I do not see any mention of surgery in the tables, etc.

*Thank you for recognizing and pointing out this error. In this study, we did not collect data regarding surgical therapy, but only systemic chemotherapy, palliative care, and mortality. We have corrected this in the Methods section.*
